# The impact of Irritable Bowel Syndrome on health-related quality of life: a Singapore perspective

**DOI:** 10.1186/1471-230X-12-104

**Published:** 2012-08-09

**Authors:** Yu Tien Wang, Hwee Yong Lim, David Tai, Thinesh L Krishnamoorthy, Tira Tan, Sylvaine Barbier, Julian Thumboo

**Affiliations:** 1 Department of Gastroenterology and Hepatology, Singapore General Hospital, Singapore; 2 National Cancer Center, Singapore; 3 Duke-NUS Graduate Medical School, Singapore; 4 Department of Rheumatology & Immunology, Singapore General Hospital, Singapore

## Abstract

**Background:**

Irritable bowel syndrome (IBS) is a common gastrointestinal disorder. The prevalence of IBS in Asian countries varies from 2.9% to 15.6%. IBS does not result in increased mortality, but is associated with psychological distress and disruption of work and sleep. Consequently, the evaluation of health-related quality of life (HRQoL) is an important outcome measure for patients with IBS since it provides a holistic assessment of the patient's emotional, social and physical function. However, some HRQoL tools can be time-consuming to apply. EQ-5D is a brief HRQoL tool which has been validated in the Western IBS population but has thus far not been used in Asia. This study was conducted to determine whether persons with self-reported symptoms that met the Rome III criteria for IBS had a poorer quality of life than those without these symptoms. We also aimed to determine which specific aspects of quality of life were most affected and whether any risk factors distinguished those with and without IBS.

**Methods:**

Self-administered questionnaires which included the Rome III diagnostic questionnaire modules for IBS and the EQ-5D questionnaire were obtained from participants of a health symposium in Singapore on 31th October 2010. IBS was diagnosed based on the Rome III Criteria. The main outcome measure was the EQ-5D index score. The relationship between the presence of IBS and the EQ-5D index score, individual dimensions of EQ-5D and demographic risk factors were examined.

**Results:**

449 completed questionnaires were analyzed. The mean EQ-5D index score for IBS was 0.739 which was a significant reduction compared to non-IBS participants [−0.11 (95% CI: -0.15 to −0.07), p < 0.001]. Multivariate analysis showed that IBS was significantly associated with younger age and higher education level. Of the five EQ-5D dimensions, IBS sufferers were significantly affected in mobility, anxiety or depression, usual activity and pain. There was a "dose related" increase in likelihood of having IBS with increased severity of pain and anxiety or depression.

**Conclusion:**

IBS sufferers have significantly poorer quality of life. Assessment of HRQoL in IBS using the EQ-5D should be considered in further studies and routine clinical practice.

## Background

Irritable bowel syndrome (IBS), a well-known functional gastrointestinal disorder, is characterized by abdominal pain, bloating or abdominal discomfort occurring in association with disturbed bowel patterns in the absence of organic causes detectable by routine investigations [1]. The prevalence of IBS in Asian countries varies from 2.9% to 15.6% [1]. While IBS does not result in increased mortality, it is associated with psychological distress and disruption of work and sleep.

Consequently, the evaluation of health-related quality of life (HRQoL) is an important outcome measure for patients with IBS since it provides a holistic assessment of the patient’s emotional, social and physical functions [2]. HRQoL is important in assessing both severity and treatment outcomes of IBS [2]. A generic evaluation of HRQoL also allows for comparisons between diverse diseases and may aid in decisions about healthcare resource allocation. The importance of HRQoL in IBS is noted by the comparable impact of diabetes and IBS on physical function, and by evidence of lower mental HRQoL in IBS than in those with chronic renal failure [3]. The American College of Gastroenterology recommends the routine screening of HRQOL in IBS patients [4] in order to holistically assess the severity and impact of the disease.

The assessment of HRQoL in IBS patients can be quite time-consuming. The Short Form 36-Item Health Survey (SF-36), a commonly used HRQoL assessment tool, may require 7–10 minutes [5], which precludes its use in routine care. Recently, a brief HRQoL tool, the EQ-5D [6], was validated in IBS on several Western samples [7]. The EQ-5D had moderate to strong associations with SF-36 in the study. While the EQ-5D has been validated across the 3 major ethnic groups in Singapore in non IBS studies [8,9], we know of no publication on its use in IBS in Asia. The EQ-5D comprises of five questions (mobility, self-care, usual activities, pain/discomfort and anxiety/depression). Each question consists of responses at 3 levels: no problems, some problems and extreme problems. There is also a separate visual analogue scale which is rated from 0 to 100.

This study was conducted to determine whether firstly, persons from a community sample of opportunity with self-reported symptoms that met the Rome III criteria for IBS [10] had a poorer quality of life than those without these symptoms. Secondly, we also aimed to determine which specific aspects of quality of life were most affected and thirdly, whether any risk factors distinguished those with and without IBS.

## Methods

This cross-sectional study consisted of obtaining the self-reported EQ-5D [11], from all participants attending the National Foundation for Digestive Diseases Symposium held at the Raffles City Convention Centre, Singapore on 31th October 2010 (estimated audience size = 850 people). This free public education symposium discussed gastrointestinal malignancies in both English and Mandarin. The symposium was advertised in Chinese and English newspapers.

The questionnaire was obtained throughout the duration of the symposium on the same day by the use of distribution and collection booths. Study investigators, who were doctors familiar with IBS and the EQ-5D, were available to answer questions and assist respondents (if needed ) in the completion of the questionnaire (provided in English and Chinese).

This study was approved by the Singhealth Centralised Institutional Review Board. The purpose of the study was detailed in the cover page of the questionnaire and consent was inferred from completion and return of questionnaires.

### Questionnaires

The questionnnaires included the Rome III diagnostic questionnaire modules for IBS [10] and the EQ-5D [11] either in English or Chinese. The questionnaire also included the participants’ age, gender, ethnic group, education level, employment status, presence of other significant illnesses and presence of significant illnesses in another family member of the participant.

IBS was ascribed based on the Rome III diagnostic criteria for IBS [10] which requires abdominal pain or discomfort in at least 3 of the previous 6 months, with 2 or more of the following criteria: [1] pain or discomfort improved after defecation; [2] pain or discomfort associated with a change in frequency of stool, or [3] pain or discomfort associated with a change in the form of stool.

HRQoL was assessed using the EQ-5D-3 L [11] which includes 2 pages of the EQ-5D descriptive system. The EQ-5D descriptive system comprises the following 5 dimensions; mobility (ability to walk), self-care (ability to wash and dress oneself), usual activities (ability to perform usual activities such as work, study, housework and family or leisure activities), pain/discomfort (experiencing pain or discomfort) and anxiety/depression (feeling anxious or depressed). Each dimension has 3 levels of responses: no problems (assigned a rating of 1), some problems (assigned a rating of 2), or severe problems (assigned a rating of 3). The respondent indicated his or her current health state by choosing the most appropriate statement in each dimension. Ratings for each dimension were then combined into a 5-digit number that describes the respondent’s health state. This 5 digit EQ-5D health state was converted into a single summary index score by inputing the 5 digit number into the EQ-5D index calculator available from the EuroQol Group [12]. In our study, the United Kingdom Value set [13] was applied as no local value set was available and because the United Kingdom value set has robust evidence. The Index score was our primary end-point (range from −0.6 to 1). A score of 1 represents a health state of full health and 0 represents a health state of death. A negative score represents a health state worse than death.

The relationship between demographic risk factors and the presence of IBS was studied using the binary logistic regression model. From the significant variables identified in the univariate analysis, we used the backward stepwise method to identify variables that were independently and significantly associated with the outcome using the likelihood ratio test. The odds ratios and their corresponding 95% confidence intervals were presented as effect size. We also repeated this modeling strategy for the various sub-domains within the EQ-5D. In addition, we used the ordinary least-squares regression model to analyse factors that were associated with the overall EQ-5D score. Once again, a backward stepwise method was used to build up the multivariate model. Tests for the appropriate assumptions for the models were carried out. Data was analysed using Stata V11.2 (Stata Corp, College Station, Tx, USA) and the level of significance was set at 5%.

## Results

464 questionnaires were collected. Of these, a total of 449 questionnaires constituted the analyzable sample. 15 questionnaires were excluded due to incomplete responses which precluded determination of their IBS status or EQ-5D index score.

We found that 198/449 (44.1%) of respondents had met the Rome III diagnostic criteria for IBS. More males had IBS (53% vs 42%, p = 0.015) but the races were equally distributed among the two groups. There was no association with having illnesses themselves or in other family members. The factors associated with IBS were younger age [adjusted OR = 0.98 (95% CI: 0.96 to 0.99)] and a higher education level, defined as the possession of a diploma, degree or post graduate qualification [adjusted OR = 1.7 (95% CI: 1.11 - 2.58), p = 0.014]. The odds for having IBS were 3.78 times greater for a person who had done post-graduate studies versus a person with a primary level education (p = 0.014). The odds for having IBS were also 1.68 times greater for a person who was employed as compared to one who was unemployed, retired, a housewife or a student (p = 0.009) (Table 1).

**Table 1:** Factors associated with IBS status

		**IBS**				**Odds ratio (univariate analysis)**		**Odds ratio (multivariate analysis)**	
		**No**		**Yes**		**OR (95% CI)**	**p-value**	**OR (95% CI)**	**p-value**
Age [Median years (IQR)]		57 (50–62)		52 (44–59)		0.97 (0.95 - 0.99)	<0.001	0.98 (0.96 - 0.995)	.012
Gender	Male	102	42%	105	53%				
	Female	143	58%	92	47%	0.62 (0.43 - 0.91)	0.015		
Race	Chinese	230	93%	179	91%				
	Indian	11	4%	11	6%	1.28 (0.54 - 3.03)	0.567		
	Malay	1	0%	2	1%	2.57 (0.23 - 28.57)	0.442		
	Others	5	2%	5	3%	1.28 (0.37 - 4.51)	0.695		
Education	Low level	135	56%	74	38%				
	High level	105	44%	121	62%	2.1 (1.43 - 3.09)	<0.001	1.7 (1.114 - 2.581)	.014
Work	Unemployed	114	47%	68	35%				
	Employed	128	53%	128	65%	1.68 (1.14 - 2.47)	0.009		
Illness in yourself	No	53	24%	44	24%				
	Yes	170	76%	136	76%	0.96 (0.61 - 1.52)	0.874		
Illness in Family	No	81	50%	83	55%				
	Yes	82	50%	68	45%	0.81 (0.52 - 1.26)	0.350		

IBS was significantly associated with four of the five EQ-5D dimensions (except “self-care”; p = 0.77). The probability of having some mobility problems for persons suffering from IBS is 2.38 times higher than the probability for the ones without IBS (p = 0.02). The probability of having some problems with "usual activity" for persons suffering from IBS is 3.63 times higher than the probability for the ones without IBS (p < 0.001). In the “pain/discomfort” dimension, the probability of having some pain or discomfort was 4.92 times higher for persons with IBS versus one without(p < 0.001) while it was 7.93 times higher for persons with IBS to experience extreme pain or discomfort as compared to persons without IBS(p = 0.002) . The probability of being moderately anxious or depressed and of being extremely anxious or depressed is also higher for persons suffering from IBS (2.39 times, p < 0.001 and 3.68 times, p = 0.037, respectively) (Table 2).

**Table 2:** Association of the 5 dimensions of EQ-5D with IBS status

		**IBS**				**Odds ratio (univariate analysis)**		**Odds ratio (multivariate analysis)**	
		**No**		**Yes**		**OR (95% CI)**	**p-value**	**OR (95% CI)**	**p-value**
		**Count**	**%**	**Count**	%				
Mobility	No Problem	236	94%	176	89%				
	Some Problems	15	6%	22	11%	1.97 (0.99 - 3.9)	0.053	2.38 ^a^ (1.15 - 4.96)	0.020
Self-care	No Problem	248	99%	195	98%				
	Some Problems	3	1%	3	2%	1.27 (0.25 - 6.37)	0.770	1.58 ^b^ (0.22 - 11.52)	0.654
Usual Activity	No Problem	234	93%	164	83%				
	Some Problems	17	7%	34	17%	2.85 (1.54 - 5.28)	0.001	3.63 ^c^ (1.85 - 7.14)	<0.001
Pain/ Discomfort	No Pain or discomfort	143	57%	40	20%				
	Some Pain or discomfort	104	41%	150	76%	5.16 (3.35 - 7.93)	<0.001	4.92 ^d^ (3.15 - 7.68)	<0.001
	Extreme Pain or discomfort	4	2%	8	4%	7.15 (2.05 - 24.97)	0.002	7.93 ^d^ (2.2 - 28.57)	0.002
Anxiety/ Depression	Not anxious or depressed	164	65%	94	47%				
	Moderately anxious or depressed	82	33%	96	48%	2.04 (1.38 - 3.01)	<0.001	2.39 ^e^ (1.57 - 3.64)	<0.001
	Extremely anxious or depressed	5	2%	8	4%	2.79 (0.89 - 8.78)	0.079	3.68 ^e^ (1.08 - 12.5)	0.037

^a^ adjusted on Age and Education (remained significant on multivariate analysis).

^b^ adjusted on Age and Education (remained significant on multivariate analysis).

^c^ adjusted on Work and Education (remained significant on multivariate analysis).

^d^ adjusted on Education (remained significant on multivariate analysis).

^e^ adjusted on Age and Education (remained significant on multivariate analysis).

The mean EQ-5D index score was 0.739 for IBS and 0.854 for non-IBS. After adjustment for age, gender and presence of significant illnesses in another family member of the participant, the effect of IBS on the EQ-5D still showed a significant difference [−0.11 (95% CI: -0.15 to −0.07), p < 0.001] (Table 3).

**Table 3:** Linear regression - Impact of IBS status on Quality of life (measured with the EQ-5D scale)

		**Coeff.**	**95% CI**	**p-value**
**Non-adjusted**	non-IBS (reference)			
	IBS	−0.114	−0.15 to −0.08	<0.001
Adjusted*	non-IBS (reference)			
	IBS	−0.109	−0.15 to −0.07	<0.001

*adjusted on age, gender and Illness in other family member (factors which remained significant on multivariate analysis).

## Discussion

This study found that persons with IBS symptoms that meet the Rome III criteria have poorer quality of life. Our cohort of community-based IBS sufferers had a mean EQ-5D index score of 0.739 which is comparable to patients who suffer from other serious and debilitating conditions such as chronic ischaemic heart disease, which was shown to have a mean index score of 0.738 in one study [14]. The prevalence rate of IBS in Singapore has previously been found to be rather high at 8.6% [15] with a clinic consultation rate of 84% [16]. These high rates and the significant negative impact contribute to a hefty disease burden and increased healthcare costs to the community.

The literature on Western populations has shown that among the five EQ-5D domains, pain/discomfort was the most adversely affected while self-care was the least affected in IBS sufferers [7]. In our study, we found that pain/discomfort was most significantly affected in the IBS sufferers as well. In addition to this, the mobility, activity and anxiety/depression domains were also significantly affected among the IBS sufferers. However, the self-care domain was not significantly affected. To our knowledge, no prior quality-of-life studies on IBS in Asia have used the EQ-5D tool. However, a study in China which used SF 36 for IBS patients showed that the domain of “physical function” was not affected [17]. It has also previously been shown that the self-care domain in EQ-5D strongly correlates with the “physical function” domain in SF 36 [7]. On the other hand, the number of respondents who reported any problems with self-care was very low [6 out of 449 patients (1.3%)]. Hence, it may not be sufficiently powered to detect any difference between the two groups.

There is a “dose-related” association between anxiety and depression with IBS. Respondents were more likely to have IBS when they had a higher degree of reported anxiety or depression. It is well-recognized that psychological disturbances are common in IBS [1] and may result in changes in perception of visceral sensations in IBS patients [18]. However, secondary psychological problems can also develop as a result of a chronic debilitating condition such as IBS. While this cross-sectional study cannot establish a causal relationship, the strong association supports recommendation that psychological disturbances should be actively explored and managed in IBS patients [1].

There was a male preponderance in the IBS group (53% vs 42%) but this was not significant on multivariate analysis. One Indian study [19] has shown a significant association of between males and IBS but most Asian studies have shown parity between the genders [20]. Our results reinforce the observations that the greatly increased risk of IBS in women reported in the West is not reflected in the Asian community [20]. The cause of this difference is unknown and warrants further investigation.

Asian studies show IBS is more prevalent in younger and more educated individuals [20]. Our findings are in line with these observations. In addition, we found IBS was also associated with employment although this difference was not significant on multivariate analysis. This can be explained by the over-representation of retired people in the unemployed group, who are significantly older than the employed group (mean age was 64 years in the unemployed group versus 49 years in the employed group, p < 0.001). The association between employment and IBS has not been extensively studied and it would be interesting to explore this association.

There are some limitations in this study. The first is that the study population is from participants in a public symposium focusing on gastrointestinal malignancy. It is likely that many of them may have significant gastrointestinal symptoms and are better educated, both factors which are associated with increased risk of IBS. Hence it is not representative of the general population of the country and data such as prevalence of IBS in this group cannot be extrapolated to the nation. Nonetheless, comparisons of HRQoL and the extent to which the individual domains are affected between IBS and non-IBS respondents are valid. The second limitation is the use of a self-reported questionnnaire for the diagnosis of IBS. Without a formal evaluation by a physician to exclude other diseases, the self-reported questionnaire may have limited accuracy for the diagnosis of IBS. It would be ideal if the conditions set forth by the regional consensus guidelines [1] were adhered to.

## Conclusion

IBS is a prevalent condition with significant negative impact on the quality of life. In our Chinese-predominant study population, the HRQoL instrument, EQ-5D, showed results and characteristics which were largely in line with Western data but with minor differences which were also observed with another HRQoL instrument in China. The EQ-5D is a brief HRQoL tool which could be considered for routine clinical application and for future studies of IBS in Asia.

## Competing interests

The authors declare no competing interests.

## Authors’ contributions

The study was jointly conceptualised by HYL, DT and YTW . TT wrote the draft for the institutional review board application which was then reviewed and submitted by YTW. YTW, HYL, DT, TT, TLK contributed to the execution of the study and acquisition of data. SB provided biostatical analysis. The manuscript was written by YTW. JT provided guidance on the methodology, analysis of the results and final critique of the paper. All authors read and approved the final article.

## Pre-publication history

The pre-publication history for this paper can be accessed here:

http://www.biomedcentral.com/1471-230X/12/104/prepub
